# [Retracted] Roles of the SNHG7/microRNA-9-5p/DPP4 ceRNA network in the growth and ^131^I resistance of thyroid carcinoma cells through PI3K/Akt activation

**DOI:** 10.3892/or.2026.9105

**Published:** 2026-03-23

**Authors:** Wanzhi Chen, Jichun Yu, Rong Xie, Tao Zhou, Chengfeng Xiong, Shuyong Zhang, Meijun Zhong

Oncol Rep 45: 3, 2021; DOI: 10.3892/or.2021.7954

Following the publication of this paper, it was drawn to the Editor's attention by a concerned reader that there appeared to be a series of internally duplicated groupings/repeated patternings of cells within various of the panels in the DPP4 row of data panels in Fig. 8B on p. 14 that could not be easily attributed to coincidence. Secondly, some of the data panels shown in the p-Akt row of data in Fig. 8B, in addition to certain of the data showing the results of EdU assay experiments in Fig. 3B on p. 8, were strikingly similar to data that had either already been submitted for publication, or which were already published, in articles in other journals that were written by different authors at different research institutes (one of which has subsequently been retracted). Furthermore, there appeared to be one instance of duplication of western blot data comparing Fig. 5K with Fig. 7, and also possible discrepancies between the actual sizes and the reported sizes of the tumours in Fig. 8A.

In view of the fact that the abovementioned data had already been submitted for publication, or were already published, elsewhere before this paper was received at *Oncology Reports*, in addition to the other problems with the data and apparent anomalies that have been identified with various of the figures, the Editor has decided that this paper should be retracted from the Journal. After contacting the authors, they accepted the decision to retract the paper. The Editor apologizes to the readership for any inconvenience caused.

